# Alum in Pertussis

**Published:** 1847-10

**Authors:** 


*Alum in Pertussis.*—Dr. Davies thus speaks of the employment of alum in pertussis:—

After a long trial, I am disposed to attach more importance to alum as a remedy in hooping-cough, than to any other form of tonic or antispasmodic. I have often been surprised at the speed with which it arrests the severe spasmodic fits of coughing; it seems equally applicable to all ages, and almost all conditions of the patient. I was formerly in the habit of taking much pains to select a certain period of the illness for its administration, and waiting until the cough had existed at least three weeks, taking care that the bowels were open, the patient free from fever, the air-passages perfectly moist, and the disorder free from complication of any bruit. A continued observation of the remedy, however, has induced me to be less cautious, and I am disposed to think that a very large amount of collateral annoyances will subside under its use. The fittest state for its administration will be a moist condition of the air-passages, and freedom from congestion, but an opposite condition would not preclude its use, should this state not have yielded to other remedies. It generally keeps the bowels in proper order, no aperient being required during its use.

The dose for an infant is two grains daily; and to older children, four, five, and up to ten or twelve grains may be given, mixed with syrupus rhœados (syrup of red poppy) and water. It is seldom disliked.—*Underwood's Diseases of Infants.*

